# Harvest and density‐dependent predation drive long‐term population decline in a northern ungulate

**DOI:** 10.1002/eap.2629

**Published:** 2022-06-23

**Authors:** Robby R. Marrotte, Brent R. Patterson, Joseph M. Northrup

**Affiliations:** ^1^ Ontario Ministry of Natural Resources & Forestry, Wildlife Research & Monitoring Section Trent University Peterborough Ontario Canada; ^2^ Environmental and Life Sciences Graduate Program Trent University Peterborough Ontario Canada

**Keywords:** *Alces alces*, Bayesian population model, density dependence, harvest management, hierarchical model, hunting, moose, predation, top‐down bottom‐up, ungulate

## Abstract

The relative effect of top‐down versus bottom‐up forces in regulating and limiting wildlife populations is an important theme in ecology. Untangling these effects is critical for a basic understanding of trophic dynamics and effective management. We examined the drivers of moose (*Alces alces*) population growth by integrating two independent sources of observations within a hierarchical Bayesian population model. We used one of the largest existing spatiotemporal data sets on ungulate population dynamics globally. We documented a 20% population decline over the period examined. There was negative density‐dependent population growth of moose. Although we could not determine the mechanisms producing density‐dependent suppression of population growth, the relatively low densities at which we documented moose populations suggested it could be due to density‐dependent predation. Predation primarily limited population growth, except at low density, where it was regulating. After we simulated several harvest scenarios, it appeared that harvest was largely additive and likely contributed to population declines. Our results highlight how population dynamics are context dependent and vary strongly across gradients in climate, forest type, and predator abundance. These results help clarify long‐standing questions in population ecology and highlight the complex relationships between natural and human‐caused mortality in driving ungulate population dynamics.

## INTRODUCTION

Globally, large herbivores are important cultural and economic resources that shape the function and structure of landscapes (Trouwborst, 2019). Thus, these species are among the most intensively managed (Ripple et al., 2015). In areas where they are hunted, their sustainable harvest is essential for balancing healthy ecosystems and social and cultural practices, yet many populations are declining and face the risk of extinction (Di Marco et al., 2014; Erb & Boyce, 1999). Further, climate and land‐use change are amplifying the effects of predation, harvest, competition, and disease on these species (Ripple et al., 2015). Understanding how key population drivers interact to influence population growth is critical to the management and conservation of these species under continued global change. However, concurrent analyses of top‐down and bottom‐up processes in terrestrial systems over periods that are long enough to capture complex population dynamics remain rare, limiting inference about the relative importance of potential drivers (Vucetich & Peterson, 2004).

Although intraspecific competition is thought to be the primary factor regulating populations of ungulates, predation can also play a key role in both limiting and regulating population growth (Ballard & van Ballenberghe, 2008; Patterson & Power, 2002; Sinclair, 2003). Further, predation can be strongly density‐dependent, with predation regulating populations at low densities (Messier, 1994; Messier & Crête, 1985; van Ballenberghe & Ballard, 1994) but becoming less important at higher densities because of predator satiation (Jost et al., 2005; Messier, 1995; Vucetich et al., 2002; but see also Eberhardt et al., 2003). Where ungulates are subject to harvest, predation can also be important in regulating populations depending on population size relative to carrying capacity and whether the harvest is additive or compensatory (Boyce et al., 1999; Kokko & Lindström, 1998; Timmermann & Rodgers, 2017). In areas where predator populations have been depleted, other factors such as disease and parasites can become major limiting factors (Jones et al., 2017; Musante et al., 2010).

Persecution of predators, along with large‐scale habitat loss and climate change, has altered ecosystems to the point where the factors limiting and regulating large ungulate populations are now substantially different from those under which species evolved (Child et al., 2019; Dublin & Ogutu, 2015; Pierce et al., 2012), complicating our understanding of contemporary population dynamics. Considering that climate and land‐use change are projected to continue, assessing the putative drivers of population dynamics over gradients in limiting and regulating factors can provide a more complete understanding of context dependency while simultaneously offering insight to how ungulate populations will likely respond to future changes.

Here, we assessed the relative strength of intraspecific competition, predation, harvest, and disease using 55 replicated populations of moose (*Alces alces*) across nearly 1 million km^2^ and over 20 years. At the extremities of their range, moose are thought to be limited in their distribution to the north by severe winter conditions and to the south by heat stress (Monteith et al., 2015; Murray et al., 2006; Sivertsen et al., 2012). Many populations along the southern edge of their distribution in North America are in decline (Broders et al., 2012; Timmermann & Rodgers, 2017; Wattles & DeStefano, 2011). In areas with intact predator populations, predation, primarily by gray wolves (*Canis lupus*) is thought to be the primary factor limiting population growth (Ballard et al., 1987; Ballard & van Ballenberghe, 2008; Messier, 1994).

In the southern parts of moose range, extirpation of wolves has altered predator–prey dynamics, and parasites such as the winter tick (*Dermacentor albipictus*), meningeal worm (*Parelaphostrongylus tenuis*), and giant liver fluke (*Fascioloides magna*) appear to play a substantial role in the population dynamics of some populations (Ditmer et al., 2020; Jones et al., 2017; Musante et al., 2010). Although management authorities typically employ sustainable harvest strategies, hunting can also be a major mortality source for moose (Crête, 1987; Solberg et al., 1999; Timmermann & Rodgers, 2017). Predation by bears may also be a significant source of mortality for calves (Ballard & van Ballenberghe, 2008; Gasaway et al., 1992). Finally, warmer and shorter winters have facilitated the northward expansion of the white‐tailed deer (*Odocoileus virginianus*), potentially leading to apparent competition and increases in the shared parasites that deer carry (Barber‐Meyer & Mech, 2016; Lenarz et al., 2010; Murray et al., 2006).

Our objective was to examine the drivers of moose population growth in Ontario, Canada. In Ontario, predator communities are largely intact across most of the moose range but exist at lower density in the south; accordingly, we hypothesized that population dynamics would be driven primarily by predation by gray wolves (Ballard et al., 1987; Ballard & van Ballenberghe, 2008; Gasaway et al., 1992) and to a lesser extent by black bears (*Ursus americanus*). We also hypothesized that intraspecific competition, parasites associated with white‐tailed deer, and hunting would all play smaller but significant roles in limiting population growth. We predicted that population growth would be negatively related to moose density due to competition for limited resources and deer density because of shared parasites. We further predicted that moose in areas with high black bear density would exhibit lower population growth rates. Finally, because moose are harvested, and because hunting is thought to be largely additive to other factors limiting population growth (Patterson et al., 2013), we predicted that harvest rates would be negatively associated with population growth rate.

## MATERIALS AND METHODS

### Study area

**FIGURE 1:**
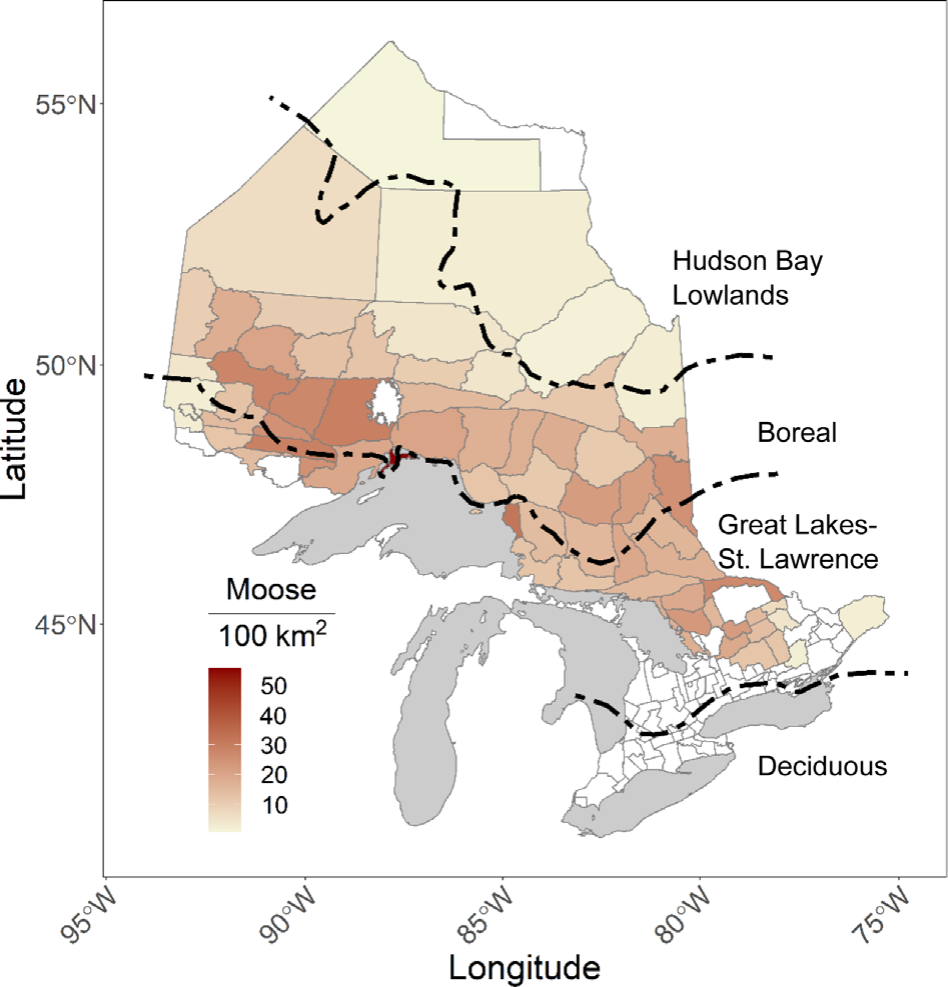
Moose density estimates for Wildlife Management Units (WMUs) where moose are hunted in Ontario, Canada. The 2020 population WMU estimates were gathered from the Government of Ontario's Wildlife Policy Branch (www.ontario.ca/page/moose‐population‐management). Boundaries of major forest regions (Rowe, 1972) are shown in dashed lines.

The study took place throughout most of the distribution of moose in Ontario, Canada (Figure 1). Moose are monitored and managed within Wildlife Management Units (WMUs). In 2019, there were an estimated 91,000 moose in the province, with the management objective for the next 10 years being to maintain the number of moose in the province between ~78,000 and 120,000 (ontario.ca/page/moose‐ontario). In 2020, licensed hunters harvested ~4000 moose. Subsistence harvesting also occurs by Indigenous peoples throughout moose range in Ontario. Both gray wolves and black bears inhabit most moose range in the province, with the smaller Algonquin wolf (*Canis cf. lycaon*) present in the southern extent. White‐tailed deer also overlap with moose, with the highest densities in the Great Lakes‐St. Lawrence (GLSL) forest region in the southeastern and extreme western parts of the province.

### Data

To examine factors driving moose population dynamics, we obtained data on moose, white‐tailed deer, predators (canids and bears), hunter effort, and harvest. In the 69 WMUs where moose were hunted, abundance was estimated from standardized aerial surveys undertaken from December to mid‐February at least once every 4 years since 1975 (McLaren, 2006). During each survey, information was recorded on the age class (calf or adult) and sex of the observed moose (Oswald, 1997). Generally, surveys were stratified random block surveys similar to Gasaway et al. (1986), and a few WMUs in the Hudson‐James Bay Lowlands were surveyed using systematic belt transects (McLaren, 2006).

In addition to standardized aerial surveys, each year, the Ontario Ministry of Natural Resources and Forestry sent out questionnaires to hunters that purchased a license (Appendix S1: Figure S1). Hunters were asked to enumerate the species they saw during their moose and deer hunt. These surveys provided additional data on moose, white‐tailed deer, and canids (wolves or coyotes [*Canis latrans*]) seen. Hunters also reported whether they harvested a moose and the number of days hunted. We divided the counts of animals by the total number of days spent hunting to provide a standardized average number of animals seen each day over the entire hunting season during each year for each WMU. Lastly, we obtained information on black bear density from spatial genetic capture–recapture surveys (Howe et al., 2013). These data did not vary temporally and were from surveys undertaken between 2004 and 2010.

For moose numbers reported by hunters, several factors likely influenced the probability of detecting a moose. In the questionnaire, hunters reported the number of days they spent hunting, which we used to account for variation in the effort. Detection probability was likely also influenced by visibility during the moose hunt; it might be easier to spot moose when there was snow on the ground. To account for this potential detection bias, we gathered snow depth information from weekly measurements from the Snow Network for Ontario Wildlife (OMNRF, 2020). For each year, we summed all weekly measurements collected at each station from October to late December. We then interpolated the data across our study area using ordinary kriging and calculated the average value for each WMU. All predictors of population dynamics and observation were standardized to unit variance.

### Population model

Both aerial survey and hunter reporting data sets offered insight on moose populations across the province. Aerial moose counts provided theoretically unbiased estimates of population size but had incomplete spatial and temporal coverage. Hunter surveys provided annual information, but counts were not standardized in any way and spatial coverage was uncertain. The combination of both data sets in a model‐based framework can leverage the strengths of each data set. We integrated these data sets to estimate moose numbers using a hierarchical Bayesian population model and tested hypotheses of moose population dynamics between 1999 and 2018.

We represented density‐dependent population dynamics with a linearized discrete‐time Gompertz population growth and harvest model where the mean expected postharvest population size in each year in each WMU (*N*) was given as

(1) $$N_{\mathrm{tj}}=\exp\left(\mu_{t-1j}+r_{\max}+b_{j}\times \mu_{t-1j}+\beta_{j}x_{t-1j}\right)\;-\;E_{t-1j},$$

where *μ*_*t*−1*j*_ was the natural log of the real postharvest population size of the preceeding year, *t* the year, *j* the WMU, *r*_max_ the intrinsic rate of growth from low abundance (*N* = 1), *b* the term that controlled the level of bottom‐up density‐dependent processes, and *E* the number of moose harvested, here indexed to the preceding hunting season (Dennis et al., 2006; Koons et al., 2015; Turchin, 2013). To this model we added predictors of population dynamics ***x*** and their parameter estimates **β**. Under this formulation, the breeding population observed in the winter produced the new generation in the spring. These moose were then harvested in the fall, and the remaining moose were observed in the winter before becoming the breeding population for the following year.

We considered the *r*_max_ term a species trait that represented the capacity for growth under no limitation by any factor. Consequently, we did not allow it to vary across the different management units. Further, as shown by Lebreton and Gimenez (2013), this term and *b* can be nonidentifiable, requiring one to be constrained. We chose to constrain *r*_max_ (see section *Prior distributions and model implementation*), as a result estimating it at a population level rather than for each WMU was more straightforward. We assumed *b* was influenced by local landscape productivity, accordingly we allowed it to vary across the different management units and assumed these WMU‐specific parameters arose from a normal distribution, with the parameters of this distribution representing the average effect of increasing density on growth across WMUs and the variance term representing the variance across WMUs.

Hunter survey response rates were not 100%; consequently, we did not know the true number of moose harvested from the population (*E*). We did, however, know how many moose were harvested from a subset of hunters that returned their questionnaires. Therefore, we estimated the remaining unknown number of bulls, cows, and calves harvested using a binomial submodel for each group:

(2) $$E_{\mathrm{tj}}=\sum_{k=1}^{K}E_{o_{\mathrm{tjk}}}+\text{binomial}\left(n_{\mathrm{tjk}},s_{\mathrm{tjk}}\right),$$

where $\;E_{o}$ was the total number of known moose harvested, *k* the sex and age class, n the number of tags issued minus the number of reporting hunters, s the success rate of hunters that sent back their questionnaires, and *K* the number of sex and age classes (*K* = 3; bull, cow, and calf).

We used the aerial moose counts as an approximation of the true postharvest number of moose found in a WMU that year, but when there were no aerial counts, we estimated the number of moose from the Gompertz model such that

(3) $$\mu_{tj}∼\left\{\begin{array}{c} \text{normal}\left(\log\left(N_{tj}\right),\sigma_{{\mathrm{pro}}_{j}}^{2}\right)\;w=0,\\ \text{normal}\left(\log\left({\hat{N}}_{\mathrm{tj}}\right),{\left(\frac{{\hat{\sigma }}_{\mathrm{tj}}}{{\hat{N}}_{\mathrm{tj}}}\right)}^{2}\right)\;w=1, \end{array}\right.$$

where *N* was estimated from the postharvest parameterized Gompertz process model given earlier (Equation 1). We assumed that the log number moose in a management unit each year arose from a normal distribution with Gaussian process error $\sigma_{{\mathrm{pro}}_{j}}^{2}$, *w* indicated whether an aerial survey was conducted (*w = 1*) or not (*w = 0*), $\hat{N}$ was the estimated number of moose counted by aerial survey, and ${\hat{\sigma }}_{\mathrm{tj}}$ was the standard error during the aerial survey undertaken in WMU *j* during year *t*. We linearized these values using the delta method by log transforming the count and transformed the standard error by dividing it by the counts.

In general, the estimates from the moose aerial surveys served to scale the Gompertz population model to the appropriate magnitude of moose abundance, while allowing for a consistent data set across the entire time series. Also, process error was only estimated in years without moose aerial inventories, since we assumed that the aerial inventory had high detectability of moose.

We then tested the influence of covariates by incorporating them as additive effects on the linearized Gompertz model, where each $\beta_{j}$ was considered a WMU‐specific vector of coefficients estimated for the matrix of covariates expected to influence the population growth rate of moose. Specifically, we included the number of canids and deer observed during the fall hunting season and an interaction between the number of canids and the number of moose. Like the density dependence term *b*_*j*_, we allowed these parameters to vary across the different WMUs because these factors vary across moose populations. These parameters were assumed to arise from normal distributions whose means represented the average effect of covariates in WMUs across the province. We also allowed the effect of canids to vary as a function of the density of black bears in the unit because there were no available temporal data of black bear density, making this term an overall predation term, such that

(4) $$\beta_{{\text{predators}}_{j}}=\beta_{{\text{canids}}_{j}}+\beta_{\text{bears}}{\text{Bear Density}}_{j}$$

In the observation model for the number of moose seen, we modeled the moose counted by hunters in unit *j* during year *t* (*y*_*tj*_) with a Poisson model with observation covariates and an observation‐level random error term to account for overdispersion:

$$y_{\mathrm{tj}}∼\text{Poisson}\left(\lambda_{\mathrm{tj}}\right)$$

$$\log\left(\lambda_{\mathrm{tj}}\right)=\alpha_{j}+\vartheta_{j}z_{\mathrm{tj}}+\log\left(\exp\left(\mu_{t+1j}\right)+\;E_{tj}\right)+\eta_{\mathrm{tj}}$$

(5) $$\eta_{\mathrm{tj}}∼\text{normal}\left(0,\sigma_{\mathrm{obs}}^{2}\right)$$

where α_*j*_ was the intercept for each WMU and represented the nature log of the average proportion of the true number of preharvested moose (exp(*μ*_*t+*1*j*_)+E_*tj*_) observable in that year and unit, **ϑ**_***j***_ was a vector of coefficients estimated for each covariate in ***z***_***tj***_, and η_*tj*_ was a normal observation error term added to account for possible overdispersion. We used the number of days hunted and the fall snow depth index as observation covariates. In addition, we allowed α and **ϑ** to vary across the different WMUs and estimated them hierarchically such that they arose from a normal distribution with the mean representing the average effect of covariates across WMUs.

### Prior distributions and model implementation

We chose vague priors for means and standard deviations for the distributions on **β**, α, and **ϑ**. We also chose vague priors for σ_pro_ and σ_obs_. We used an informative prior for *r*_max_ that was originally estimated by Ruprecht et al. (2020) with a mean of 0.304 and stantard deviation of 0.08 that was calculated from previous studies that provided estimates of *r*_max_ (for more details about priors used in the model see Appendices S2 and S3).

We used a moderately informative prior for the population‐wide density dependence term *b*. We followed the approach used for plains bison (*Bison bison bison*) in Koons et al. (2015) and later used for moose in Ruprecht et al. (2020). They used a mean of 0 and truncated the distribution at −2 and 2 to exclude unrealistic effects of density dependence on population growth in the Gompertz model. Lebreton and Gimenez (2013) showed that population dynamic models, such as the one we employed, required informative priors to make meaningful inferences due to the tradeoffs between density dependence and *r*_max_ that can otherwise occur. As mentioned previously, we allowed *b* to arise from a normal distribution and chose vague priors for the mean and variance of this distribution. For the WMU‐level dependence terms, we informed these using the population‐level hyperprior.

We used an informative prior for the initial number of moose in 1999 for each WMU. We used the estimates and standard errors of aerial surveys undertaken in 1999, or, if there were no surveys, we used the closest counts in time 3 years before 1999.

### Model implementation

We used Markov chain Monte Carlo (MCMC) simulations in JAGS (version 4.3.0) (Plummer, 2018) through the R package jagsUI (version 1.5.1) (Kellner, 2019) to sample the posterior distribution of the parameters of interest. We ran three chains of two million iterations with the first 100,000 discarded as burn‐in with an adaptation of 100,000 iterations, and we thinned chains to retain every 200th simulation. This left us with 30,000 iterations, which we then used to diagnose convergence by confirming that the Gelman–Rubin diagnostic ($\hat{R}$) for each parameter was <1.1 (Gelman & Rubin, 1992). We also examined visually each parameter with trace plots. For all our analyses we used the R statistical language (version 3.6.3) (R core Team, 2020).

### General statistics

We determined any significant changes in growth rate (λ) for each wildlife management unit by first summing the preharvest moose numbers for each management unit in 2017–2018 and 1999–2000 using the estimated posterior distributions. We then calculated the average growth rate for each management unit and used quantiles to determine whether there was strong evidence for population change by building a 95% credible interval and verifying whether it overlapped λ = 1. We also calculated the estimated average proportion of moose harvested from each management unit for each year between 1999 and 2018 using estimated harvest numbers and estimated moose population size.

We could not directly compare bottom‐up and top‐down population vital rates (*r*_max_, *b*, β_canids_, β_canids *x N*_, Harvest rate, β_bears_, and β_deer_) because most occurred on different scales, so we used elasticity to determine their relative importance (Koons et al., 2007, 2015). Using our process model, we projected the equilibrium abundance of moose for each Bayesian simulation at mean covariate values and mean harvest rate. We did the same at low (2.5th percentile) and high (97.5th percentile) harvest rates. We then measured the relative effects of these parameters on their equilibrium abundance by changing each parameter individually by 1%, 5%, or 10% and computed elasticities.

## RESULTS

**FIGURE 2:**
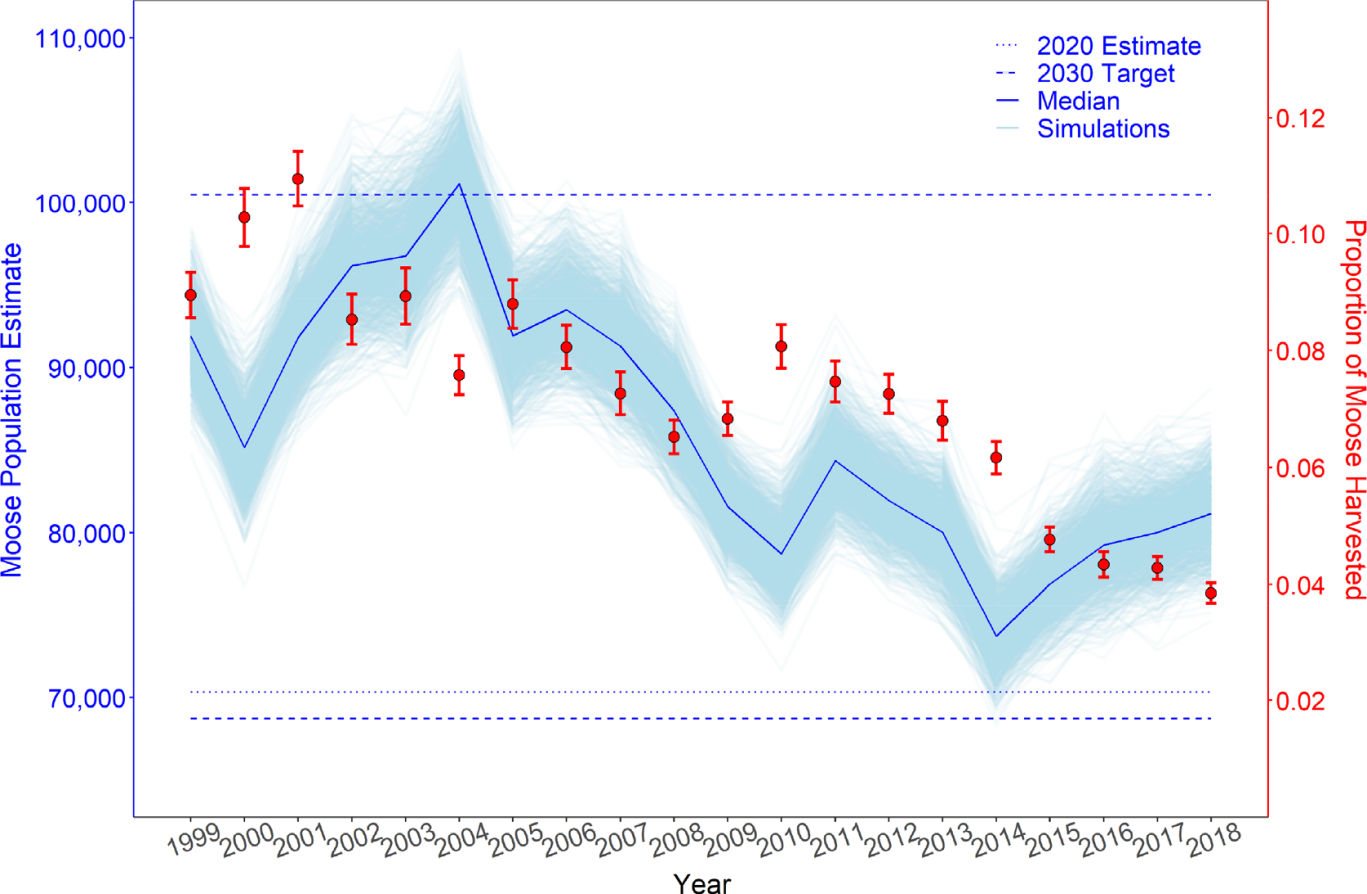
Preharvest population numbers and proportion of moose harvested in Ontario, Canada for the period 1999–2018. Moose population estimates are in blue, and the proportion of moose harvested is in red. The light blue lines are a subset of 1000 simulations randomly selected from a set of 30,000 Bayesian simulations. The 2020 estimate and 2030 population targets were gathered from the Government of Ontario's Wildlife Policy Branch (www.ontario.ca/page/moose‐population‐management).

We estimated moose abundance in 55 of 69 WMUs between 1999 and 2018. We did not include 14 units because they had <2 aerial surveys and their inclusion precluded model convergence. The remaining 55 units were surveyed ≥2 times (7 units), at most 7 times (4 units), but at a median rate of five times between 1999 and 2018. The hunter survey response rate was on average 74.1% and ranged between 50.8% and 80.3%, with >70% reporting in all but 2 years. We compared the per‐capita number of moose observed by hunters in the fall and moose counted during the aerial surveys in the subsequent winter using the raw numbers, and Pearson's correlation coefficient was 0.714. Using a log transformation of both, the Pearson's correlation coefficient was 0.772. During the period analyzed, we estimated that WMUs contained between 68,350 and 109,308 moose during the preharvest period, whereas the average proportion of animals harvested/year varied between 0.035 and 0.113 of the provincial population (Figure 2). There was a general decline in moose abundance provincially between 2004 and 2014, but numbers increased slightly thereafter. Meanwhile, the proportion of moose harvested by licensed hunters declined over the entire 20‐year period.

**FIGURE 3:**
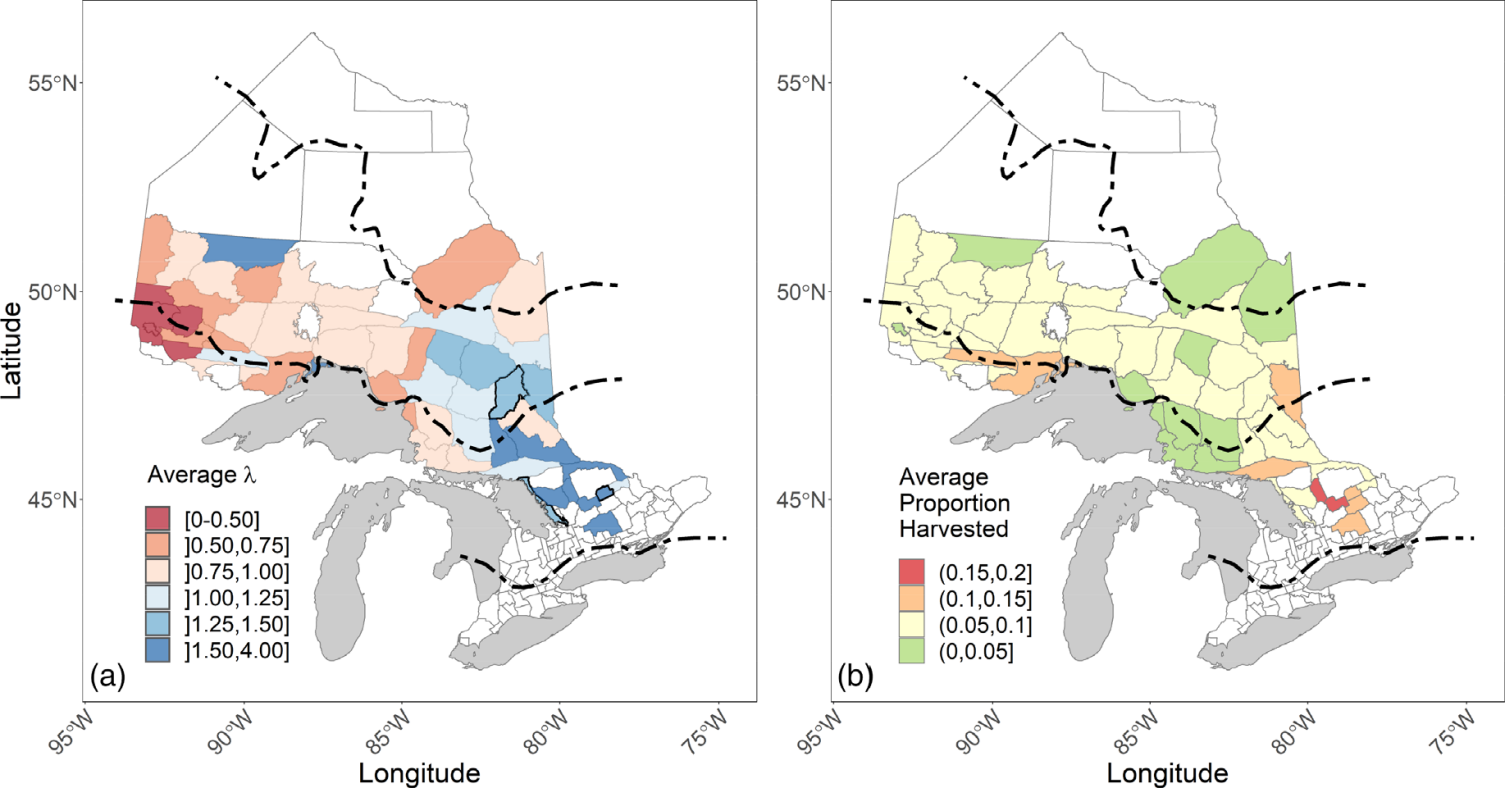
(a) Estimated average growth rate and (b) proportion harvested of moose for 55 Wildlife Management Units (WMUs) in Ontario, Canada for the period 1999–2018. (a) Population growth rate. For each Bayesian simulation, we calculated λ (*N*_2017_ + *N*_2018_)/(*N*_1999_ + *N*_2000_) and then calculated the average λ. We determined significance using quantiles and verified whether the 95% Bayesian credibility intervals overlapped 1. The three WMUs with a dark contour were the only units that did not have a significant growth rate between these years. (b) Average proportion of moose harvested. We calculated the mean proportion of moose harvested for each management unit from all 30,000 Bayesian simulations.

During these 20 years, there was strong evidence for change in moose numbers in 52 out of 55 management units (Figure 3a). Moose numbers generally declined in the northwestern portion of the province, whereas numbers in the northeast and southeast declined initially but increased in recent years. There were some notable spatial patterns in harvest (Figure 3b). In general, the units that were located near urban areas (many in the south) had the highest average proportion of moose harvested.

**FIGURE 4:**
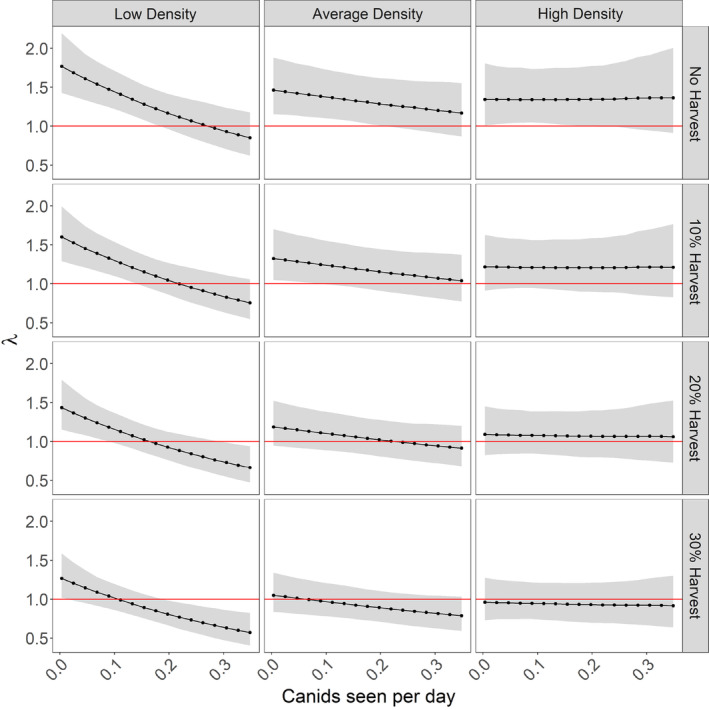
Moose population growth predictions for several scenarios of predator abundance, harvest proportion, and moose population density for an average wildlife management unit in Ontario, Canada for the period 1999–2018. Population‐level Bayesian predictions for λ for an increasing number of canids seen per day across three population levels and four harvest regimes. Moose numbers within an average‐sized wildlife management unit of 9409 km^2^: low = 100, average = 1600, and high 6000 moose, with respective densities of 1.1, 17.0, and 63.8 moose per 100 km^2^. Harvest regimes were the proportion of moose harvested from the preharvest population. Predictions were calculated with all other parameters fixed at their mean. The gray band is the 95% Bayesian credibility interval and was estimated from the quantiles of the posterior distributions

We estimated several parameters that represent predictors of moose population dynamics (Table 1). For bottom‐up population‐wide parameters, we found that *r*_max_ was higher ($\hat{\mu_{r_{\max}}}$ = 0.487, $\hat{\sigma_{r_{\max}}}$ = 0.066) than our informative prior ($\mu_{r_{\max}}$ = 0.304, $\sigma_{r_{\max}}$ = 0.08). There was also significant negative density‐dependent population growth (μ = −0.058, σ = 0.010). For top‐down population‐wide parameters, population growth was negatively associated with the number of canids observed during the hunting season (μ = −0.195, σ = 0.068). We also found strong evidence of density dependence in this term (μ = 0.023, σ = 0.010), with the effect weakening with increasing numbers of moose regardless of the level of harvest (Figure 4). Moose harvest seemed to only have an impact on population growth at low to moderate moose densities. The influence of hunting and predation both decreased with moose abundance except at very high harvest rates, far above what was implemented in practice (>0.3 or 30% of moose harvested). Lastly, we found a weak negative effect of bear and deer abundance on moose population growth at the provincial scale (Table 1).

**TABLE 1:** Population‐level parameter estimates for the Gompertz population and moose observation models for 55 Wildlife Management Units in Ontario for 1999–2018

Parameters	Mean	Median	σ	Lower 95% BCI	Upper 95% BCI	$\hat{R}$	Effective sample size	*f*
*r*_max_	**0.487**	0.486	0.066	0.358	0.618	1.000	7852	1.000
*b*	**−0.058**	−0.058	0.010	−0.077	−0.040	1.000	10,897	1.000
β_canids_	**−0.195**	−0.196	0.068	−0.330	−0.061	1.001	2544	0.998
β_canids *x N*_	**0.023**	0.023	0.010	0.003	0.042	1.001	2199	0.987
β_bears_	−0.013	−0.014	0.016	−0.044	0.018	1.000	17,274	0.808
β_deer_	−0.022	−0.022	0.013	−0.047	0.003	1.000	30,000	0.956
γ_α_	**−0.445**	−0.447	0.076	−0.591	−0.293	1.000	30,000	1.000
γ_hunt−days_	**0.734**	0.731	0.065	0.615	0.872	1.000	15,544	1.000
γ_snow_	**0.028**	0.028	0.008	0.012	0.045	1.000	30,000	0.999

*Note*: Values in bold are population effects where the lower and upper 95% Bayesian credibility intervals (BCIs) did not overlap 0. The 95% BCIs were estimated from the quantiles of the posterior distributions. $\hat{R}$ is the Gelman–Rubin diagnostic, and f is the proportion of the posterior with the same sign as the mean.

We compared the relative influence of all bottom‐up and top‐down parameters by calculating their elasticities at three levels and three harvest regimes (Table 2). We found that increasing five out of seven of our population‐level parameters induced change in moose numbers from equilibrium in all three harvest regimes. The intrinsic rate of growth (*r*_max_) had the largest elasticity, followed by density dependence (*b*) and both canid terms (β_canids_ and β_canids *x N*_). Other than *r*_max_, the elasticities for these parameters were comparable in magnitude because their 95% Bayesian credible intervals overlapped. Density dependence (*b*) and the average effect of canids ($\mu_{\beta_{\text{canids}}}$) were both negative, whereas canid density dependence was positive ($\mu_{\beta_{\text{canids}\times N}}$). The elasticity for moose harvest rate was smaller than for these other population drivers except at high harvest rates. When we calculated elasticities at an increased harvest rate, however, the elasticity for harvest was similar in magnitude to density dependence and greater than the density‐dependent effect of canid abundance. The deer (β_deer_) and bear (β_bears_) terms induced no substantial change in the number of moose.

**TABLE 2:** Elasticities from mean equilibrium abundance of moose populations to proportional changes (δ) in all six of our Gompertz population‐level parameters relative to population‐wide mean conditions

Parameters	Average harvest			Lower harvest			High harvest		
	δ = 1%	δ = 5%	δ = 10%	δ = 1%	δ = 5%	δ = 10%	δ = 1%	δ = 5%	δ = 10%
*r*_max_	**0.831**	**0.845**	**0.864**	**0.776**	**0.789**	**0.805**	**0.969**	**0.989**	**1.015**
*b*	**−0.409**	**−0.405**	**−0.401**	**−0.475**	**−0.470**	**−0.464**	**−0.243**	**−0.241**	**−0.239**
β_canids_	**−0.406**	**−0.402**	**−0.398**	**−0.406**	**−0.402**	**−0.398**	**−0.407**	**−0.402**	**−0.398**
β_canids *x N*_	**0.333**	**0.336**	**0.339**	**0.388**	**0.392**	**0.396**	**0.194**	**0.196**	**0.197**
Harvest	**−0.073**	**−0.072**	**−0.072**	**−0.010**	**−0.010**	**−0.010**	**−0.231**	**−0.229**	**−0.228**
β_deer_	−0.264	−0.262	−0.260	−0.264	−0.262	−0.260	−0.265	−0.262	−0.260
β_bears_	−0.002	−0.002	−0.002	−0.002	−0.002	−0.002	−0.003	−0.002	−0.002

*Notes*: We also included conditions where the harvest rate was low (2.5th percentile) or high (97.5th percentile) and all other population‐wide parameters were at their mean. Values in bold are population effects where the 95% Bayesian credibility intervals (BCIs) did not overlap 0. The 95% BCIs were estimated from the quantiles of the posterior distributions.

**FIGURE 5:**
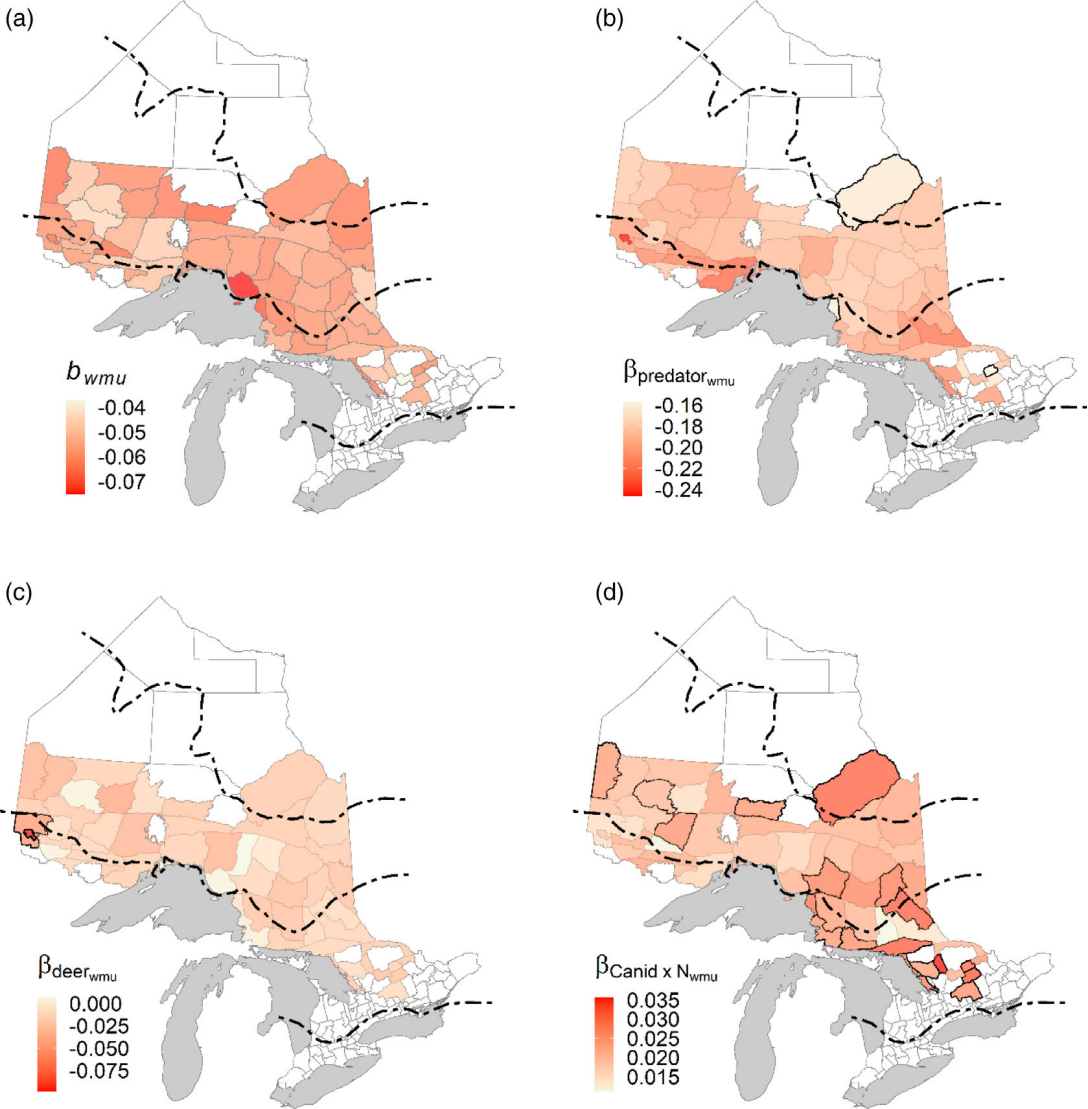
Mean parameter estimates for wildlife management unit in Ontario, Canada for the period 1999–2018. (a) Density‐dependence estimates; all units had significant negative density dependence. (b) Predator parameter estimates that include the additive effect of both canids and bear. The three wildlife management units outlined in black did not have a significant predator effect at the unit level. (c) Deer parameter estimates. Despite being nonsignificant at the population level, two wildlife units in the west had significant effects (outlined in black). (d) Predator density‐dependence estimates; despite being significant at the population level, only 18 units (outlined in black) had significant predator density dependence at unit level

Our model also allowed us to determine spatial patterns in density dependence ($b_{\mathrm{WMU}}$), predators ($\beta_{{\text{predators}}_{j}}$, including the effect of canids and bears), deer ($\beta_{{\text{deer}}_{j}}$), and canid density dependence ($\beta_{\text{canids}\times N_{j}}$) because these parameters were also estimated at the WMU level (Figure 5). All units exhibited significant negative density dependence (Figure 5a). Moose exhibited the largest magnitude of negative density dependence in a unit situated on the northeastern shores of Lake Superior. Conversely, there were also a few areas in the south and a cluster in the northwest that had values of *b* close to 0, indicating little density dependence. There were no obvious spatial patterns for the predator parameter estimates at this level of the model, but one unit in the northwest had a comparably large negative estimate (Figure 5b) and two other units showed no evidence of significant predator effects (outlined in black in Figure 5b). Only two units had significant deer parameter estimates, and both were clustered in the northwest (Figure 5c). Finally, 18 units (≈ 33%) had significant positive density‐dependent predator terms (outlined in black), but there did not seem to be any apparent spatial pattern (Figure 5d).

## DISCUSSION

Untangling the influence of top‐down versus bottom‐up effects on populations is fundamental to understanding the ecology of ungulates (Hairston et al., 1960; Messier, 1991, 1995; Sinclair et al., 2003) and is critical for effective conservation and management. Over the last 20 years, moose in Ontario at high population density appeared to be regulated by intraspecific competition, with predation by canids playing a limiting role. Wolf predation appeared to slow the population growth of moose but did not ultimately depress the numbers across most observed densities of wolves. However, when moose density was lower, and our index of wolves was well above average, predation likely played a regulating effect, leading to population decline and potentially suggesting a stable lower density equilibrium (i.e., a predator pit, sensu Gasaway et al. [1992]) in the presence of high wolf density. Past research has likewise suggested the potential for strongly density‐dependent predation in ungulate systems (Crête & Courtois, 1997; Serrouya et al., 2015; van Ballenberghe & Ballard, 1994). Our data were collected from 55 management units across a large spatial extent with strong gradients in putative limiting and regulating factors. This replication provided a robust assessment of these factors and strong evidence for the existence of density‐dependent predation. These dynamics have fundamental implications for our understanding of ungulate population dynamics under contemporary trophic assemblages and suggest the potential for human‐caused mortality (i.e., harvest) to induce population declines in ungulates in areas with healthy predator populations.

### Cause of population decline

**FIGURE 6:**
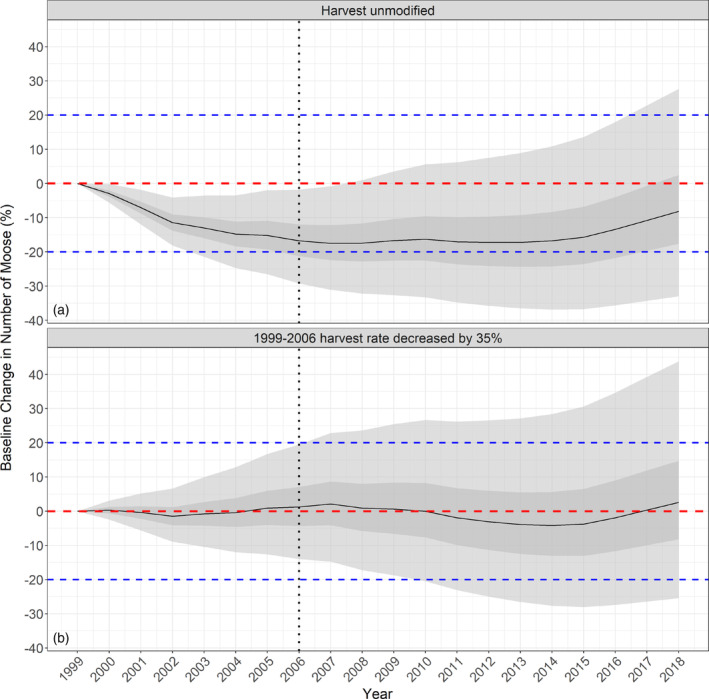
Simulated moose population dynamics for different values of harvest for an average wildlife management unit in Ontario, Canada for the period 1999–2018. Simulations were obtained from the Gompertz model using the posterior median initial population size and provincial parameters (*r*_max_, *b*, and covariate effects) at average harvest rate of each year and average covariate levels. (a) Average harvest rate unmodified from that estimated in the model. (b) Harvest decreased by 35% for the 1999–2006 period. The red dashed line is the baseline number of moose estimated in 1999. The blue dashed line represents a 20% increase or decrease from the baseline number of moose. The black dotted line separates the early high harvest period between 1999 and 2006, and this is the period where harvest was modified in the simulation, with harvest unmodified after this time point. The dark gray band is the interquartile range, whereas the light gray band is the 95% Bayesian credibility interval. Both intervals were estimated from the quantiles of the posterior distributions.

The number of moose in our study system declined by approximately 20% between 2004 and 2014. Concurrently, harvest rates were reduced annually through tag reduction. At the outset, the provincial moose population was experiencing a period of growth (Timmermann & Rodgers, 2017) and harvest exceeded 10% during some years, with certain management units experiencing even greater harvest. Harvest very likely contributed to the observed decline, making moose more susceptible to regulation by predation as wolf numbers lagged behind the decline of prey (e.g., Mech et al., 2018; Messier, 1994). When we simulated population dynamics using our posteriors, we found that the average population would decline under observed harvest for the first 14 years before increasing slightly for 6 years (Figure 6a). However, if harvest had been decreased by 35% at the beginning of the initial decline, the overall population would have been stable (Figure 6b). These simulations, in addition to our elasticity analysis, suggest that harvest played a critical role in population decline and that management was not sufficiently responsive to counteract these declines.

These population declines may take decades to reverse under current harvest management strategies. This is particularly true in areas with many wolves, where our results suggest that harvest could have pushed populations to a lower‐level equilibrium where they are now regulated by wolf predation. Positive population growth in the southern part of the province and mostly negative growth in the north, where there are more wolves, constitute further evidence that wolf predation impeded the ability of populations to compensate for high harvest and suggest that harvest was largely additive in the presence of wolves (Boertje et al., 2009; Gasaway et al., 1992; Nilsen et al., 2005).

These results provide context for our understanding of contemporary ungulate population dynamics globally. In much of the world, large predators are in decline (Ripple et al., 2014), leaving humans as a dominant driver of population dynamics for many species of ungulates (Ripple et al., 2015). Ungulate populations can sustain relatively high rates of human‐caused mortality, but these frequently occur in areas of reduced predation pressure (Gasaway et al., 1992; Reynolds & Tapper, 1996; Seip, 1992; Skogland, 1985; Strand et al., 2012; Toïgo et al., 2008). We were able to examine how ungulate populations responded to harvest across a gradient of predation pressure. Our results suggest that in jurisdictions with intact predator populations, human‐caused mortality has a strong potential to initiate population declines. These results have further implications globally for managed populations of ungulates, particularly where large carnivores are recolonizing areas from which they had been extirpated. Human‐caused mortality will need to be monitored closely in such areas, or lower densities of ungulates may need to be accepted (e.g., Nilsen et al., 2005).

### Density‐dependent processes

Our models suggest that the influence of predators on moose populations was density‐dependent at low to moderate densities but had only a weak influence on population growth at high moose density, likely owing to the asymptotic relationship between predator kill rate and prey density (Jost et al., 2005; Messier, 1994; Serrouya et al., 2015; Wilmers et al., 2007).

Similar to the analysis by Brown, (2011) on the same population but a different time period, we also found evidence suggesting strong negative density dependence at both broad and fine scales. Density dependence in ungulates originates from changes in population vital rates in response to population status relative to carrying capacity (Bowyer et al., 2014; Messier & Crête, 1984) and is a ubiquitous feature of animal systems (Sibly et al., 2005; Turchin, 1995, 1999). The apparent density dependence that we documented may suggest that there was forage competition in our moose populations. However, most populations we studied were at considerably lower densities than those where previous studies found no effect of density on indices of moose physical condition or range conditions (Crête & Jordan, 1982; Messier & Crête, 1984). Further, Crête (1989) suggested that the carrying capacity for moose in the boreal forest normally exceeds 2 moose per km^2^, several times higher than exhibited for any of the populations we studied. Thus, we find it unlikely that, at these densities, intrinsic density dependence played a large role in regulating moose populations. Alternatively, much of the apparent density dependence in moose population growth may have been attributable to a strong wolf functional response at low to moderate moose abundances. The functional response describes how the number of prey consumed per predator varies with prey density (Holling, 1959; Solomon, 1949). It is likely that there is a strong functional response by wolves to increasing moose abundance in our system (Messier, 1994; Messier & Crête, 1985; Serrouya et al., 2015). Messier (1994) demonstrated that as moose abundance increases, the functional response becomes increasingly important, and we find this mechanism to be the most likely in our system considering the low density of moose. Ultimately, a more detailed study is needed to disentangle intrinsic density dependence from regulation by wolves.

### Implications for wildlife management

Large ungulates play an important socioeconomic and cultural role in many communities, and as such, our results have ramifications for their management. Specifically, moose and other large ungulate populations cannot be managed similarly across all parts of jurisdictions that span large gradients in climate, forest type, and predator abundance. Although population fluctuations may be inevitable, harvest‐induced instability could be reduced by taking different approaches to low‐ and high‐density populations in accordance with their population trajectories. In low‐density areas, and particularly where there is a significant decline under way, predation may preclude any sustainable harvest. However, in high‐density populations, there may be additional hunting opportunities available, though care must be taken to not reduce these populations past the threshold density where predation becomes regulating. The abundance of moose below which predator regulation occurs remains to be resolved (e.g., Messier, 1994), but care needs to be taken in areas with intact predator populations. Harvest can be an additive mortality source (Gehr et al., 2018), and our results suggest that it might be relatively easy to tip ungulate populations past the point where predation can become regulating. Similarly, our results also imply that such dynamics can result from other sources of mortality that are both density‐independent and additive.

Although at the scale of the entire province of Ontario the moose population declined during our study, there was substantial variation in population trajectories at the WMU level. Most of the units that saw consistent declines were in the northwest part of the province, and two of these were the only units where our index of deer showed a strong and consistent negative relationship with moose population growth. This relationship could have been caused by the higher prevalence of meningeal worms in areas where deer were present. Our results corroborate those of moose populations studied in the same and nearby jurisdictions (Barber‐Meyer & Mech, 2016; Ranta & Lankester, 2017). If moose are a priority for management in these units, efforts should be made to keep deer abundance as low as possible. Although deer abundance did not affect moose population growth across much of the province, with climate change we can expect that deer density will increase in contact zones between the species (e.g., Kennedy‐Slaney et al., 2018). Consequently, an increase in moose mortality due to direct or indirect effects related to white‐tailed deer is likely unless proper management strategies are implemented to reduce contact.

### Study limitations

Estimating ungulate population dynamics over long periods and broad spatial scales is challenging and fraught with uncertainty. The biggest limitation of this study is that we used aerial survey and hunter reporting data with high uncertainty. We addressed this by using a framework that attempted to properly account for much of this uncertainty and propagate it appropriately from the WMU to the provincial scale. However, there likely was unaccounted for variance, and thus the precision of some effects may have been exaggerated. Another potential limitation of our study was that, although we were able to combine two independent sources of observations, we did not include the population estimator for aerial survey data directly within an observation model. This model specification would have been more complex, and our approach of using the estimated standard error should lead to similar results, though with, perhaps, deflated variance.

Another limitation was that the information reported by hunters was reported on a voluntary basis. It is possible that hunters who did not harvest a moose were less likely to report their sightings. However, we think that the hierarchical model we used was able to reduce the impact of this bias, because predictors of moose population dynamics were estimated at the management unit level, whereas a population‐wide effect was simultaneously estimated, and we made our inference at the population level. Consequently, spatial variation in bias should have had limited impact on our results, and temporal variation within a unit should have been most important. Also, there was no reason to suspect that reporting biases within a unit would have changed substantially over time. In addition, the population dynamics in our model were matched to the aerial survey data, and these were substantially more influential on the estimated abundance in each unit. As a result, any impact of temporal variation in hunter bias (e.g., skill level, hunting opportunities) should also be diminished. Lastly, assessments of bias in Ontario hunter survey data suggested that nonreporting hunters harvest big game species at rates similar to those that report (L. Hunt, *personal communication*).

In addition, our model assumed that changes in canids seen over time by hunters within a unit were representative of changes in actual canid numbers within that unit. Unfortunately, we do not have systematic predator density estimates to verify this assumption, but moose numbers reported by hunters showed a strong correlation with moose estimated from aerial surveys, suggesting that the number of canids observed by hunters should also be correlated with canid population densities. Further, Crête and Messier (1987) determined that observations of wolves by moose hunters were highly correlated with wolf densities estimated via territory mapping of radio‐collared wolves. This suggests that our metric of canid numbers was likely a valid index of relative canid numbers.

Another limitation of this research was that imperfect detection has the potential to bias moose population estimates. This primarily occurs in aerial surveys because the probability of detecting moose monotonically declines with increasing distance and vegetation cover (Giudice et al., 2012; Peters et al., 2014). However, surveyed blocks were selected randomly each year and were stratified based on different levels of expected moose density, which should have addressed concerns about intrinsic differences in moose density across units (i.e., restratification prior to each survey should ensure surveys were conducted across the variance in moose density in a unit). In addition, Ontario's survey protocols attempt to reduce error by collecting data between 12 and 72 h after a substantial snowfall, so animals have an opportunity to leave fresh tracks (McLaren, 2006). This allowed field crews to identify and follow tracks to individual moose that would have been missed because they were located off the flight line in dense conifer cover. Some moose may stay bedded during warm temperatures or high winds; as a result, surveys are limited to conditions where moose are likely to set tracks (Oswald, 1997). However, despite these efforts, detection is not perfect and may still vary by habitat type (Bisset & Rempel, 1991; Crête et al., 1986; Peterson & Page, 1993; Thompson, 1979). Detection error likely varied among WMUs due to regional variation in forest composition; however, we have no reason to believe that forest composition, and thus detection error, changed systematically within individual WMUs over the duration of our analysis. Regional trends in forest composition may influence the interpretation of the density‐dependence parameter (*b*) across WMUs. As a result, our density‐dependence parameter was not strictly estimating forage competition and likely represented a mix of factors that were unmodeled, including habitat. Nevertheless, our inferences related to growth and predators are expected to be robust to potential bias in aerial survey estimates.

Lastly, we were not able to account for additional mortality sources that are known to be present but for which we had no data. Specifically, we did not have spatiotemporal measures of harvest by Indigenous peoples, mortality due to winter ticks, and poaching at the scale of our analysis.

## CONCLUSION

Conservation of wildlife populations requires knowledge of their general population trajectories through time in space along with insight into the factors driving these dynamics. However, this knowledge requires long‐term and spatially extensive data to account for uncertainty and to sample over the range of putative drivers. As such, long‐term data sets with consistent monitoring methods are crucial to understanding wildlife population dynamics because they provide the ability to detect changes, test general hypotheses, and monitor population recovery. Our robust design provided us with unprecedented power to understand the drivers of ungulate population dynamics and our results can be generalized to other species and geographies. This analysis provides some of the most robust evidence to date for large‐scale density‐dependent predation in ungulate–predator systems and suggests similar dynamics are likely widespread, particularly in northern ecosystems with intact predator systems. Our findings also suggest that in parts of the world where predator populations are recolonizing following broad‐scale persecution, past conservation and management strategies that aim for sustainable harvest will need to be adjusted. Furthermore, our results highlight the context dependency of drivers of ungulate population dynamics. As climate and land‐use changes alter ecosystems and trophic dynamics, this context dependency will become increasingly important, and conservation strategies for ungulates will need to be fine‐tuned locally to account for specific limiting and regulating factors.

## AUTHOR CONTRIBUTIONS

Robby R. Marrotte collected data, performed modeling work, and analyzed output data. Robby R. Marrotte, Brent R. Patterson, and Joseph M. Northrup conceived the ideas and wrote the first draft of the manuscript, and all authors contributed substantially to revisions.

## CONFLICT OF INTEREST

The authors declare no conflict of interest.

## Supporting information


Appendix S1

Appendix S2

Appendix S3

## Data Availability

Data (Marrotte et al., 2022a) are available on Dryad at https://doi.org/10.5061/dryad.2280gb5tt. Code (Marrotte et al., 2022b) is available on Zenodo at https://doi.org/10.5281/zenodo.6030027.
