# Supplementary material for: Harvest and density‐dependent predation drive long‐term population decline in a northern ungulate
Source: Ecol Appl. 2022 Jun 23;32(6):e2629. doi: 10.1002/eap.2629 (PMC9541669; doi:10.1002/eap.2629)
Supplement: Supplementary file 1 — Appendix S1 [file EAP-32-e2629-s003.pdf]

Supporting Information. Marrotte, Robby R., Brent R. Patterson, and Joseph M. Northrup. Harvest and density-dependent predation drive long-term population decline in a northern ungulate. Ecological Applications.

Appendix S1

Ontario — Ministry of Natural Resources / Ministère des Richesses naturelles

Moose Hunter Questionnaire

An early assessment of the 2003 harvest is important for the management of moose. Please take a moment to answer the questions on the attached card. In question 2, we wish to know if the game seal which came with **your** licence was used to tag a moose, **no matter who shot it.**

At the end of your hunt, please tear off the attached card and mail or deliver it to an MNR office. No envelope is required. A centralized return address is being used to reduce survey costs.

In addition to this survey, you may also be sampled in the more detailed Provincial Hunter Survey. If this occurs, we encourage you to respond to it also.

Information contained on this form is requested under the authority of the Fish and Wildlife Conservation Act S.O. 1997, and will be used for the purposes of moose management and harvest assessment. Questions about the collection of this information should be referred to: MNR, Wildlife Surveys Officer, 1350 High Falls Road, Bracebridge ON P1L 1W9 Telephone (705) 646-5541.

Printed on Recycled Stock

Ontario

Moose Hunter Questionnaire

1. Did you hunt moose in 2003? ❑ Yes ❑ No
2. Did you attach the game seal that came with **your** licence to a moose? (no matter who shot the moose) ❑ Yes ❑ No

   If yes,
   (a) What type of moose was it?
   ❑ Bull ❑ Cow ❑ Calf
   (b) In which Wildlife Management Unit was the moose shot?
   ____
   (c) On what date was the moose shot? ________
   month day

3. For each Wildlife Management Unit (WMU) in which you hunted moose in 2003, please provide the following information :

	WMU # ____	WMU # ____	WMU # ____
# days spent hunting moose			
# live moose seen while hunting moose			
# live wolves/coyotes seen while hunting moose			
# wolves/coyotes killed while hunting moose			

At the end of your hunt, please complete and mail this card or deliver it to an MNR office. Thank you for your co-operation.

Figure S1. Moose hunter questionnaire sent by mail from the Ministry of Natural Resources to hunters granted a moose tag during the 2003 hunting season in Ontario, Canada. This version of the questionnaire was used between 1999-2006. There were minor modifications of the questionnaire during 2007-2018, but questions concerning the data used in this analysis were not modified.
