# Supplementary material for: Harvest and density‐dependent predation drive long‐term population decline in a northern ungulate
Source: Ecol Appl. 2022 Jun 23;32(6):e2629. doi: 10.1002/eap.2629 (PMC9541669; doi:10.1002/eap.2629)
Supplement: Supplementary file 2 — Appendix S2 [file EAP-32-e2629-s001.pdf]

Supporting Information. Marrotte, Robby R., Brent R. Patterson, and Joseph M. Northrup. Harvest and density-dependent predation drive long-term population decline in a northern ungulate. Ecological Applications.

Appendix S2

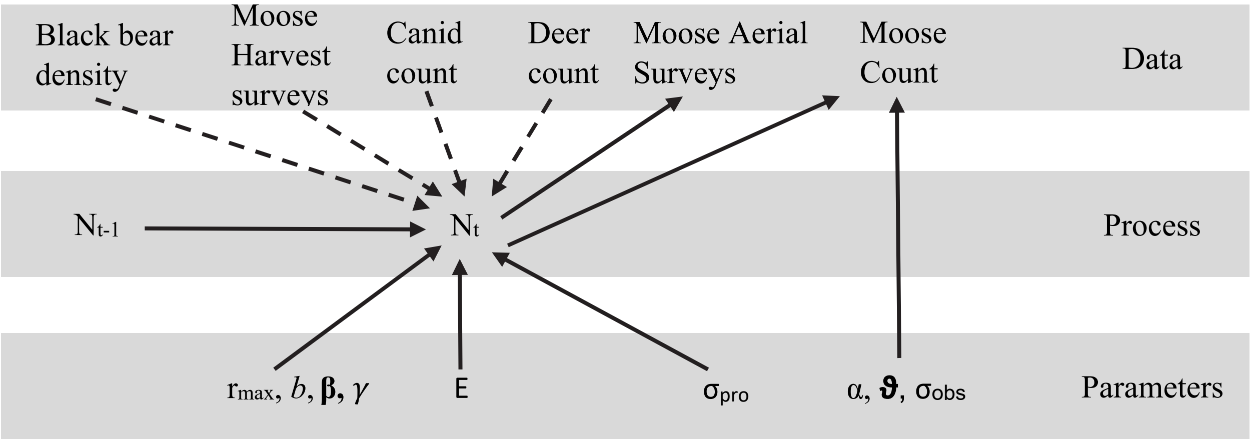

Figure S1. Directed acyclic diagram showing relationships between stochastic (solid lines) and deterministic (dotted lines) nodes for a hierarchical model describing population growth of moose in Ontario, Canada, 1999–2018.
