# Supplementary material for: Harvest and density‐dependent predation drive long‐term population decline in a northern ungulate
Source: Ecol Appl. 2022 Jun 23;32(6):e2629. doi: 10.1002/eap.2629 (PMC9541669; doi:10.1002/eap.2629)
Supplement: Supplementary file 3 — Appendix S3 [file EAP-32-e2629-s002.pdf]

Supporting Information. Marrotte, Robby R., Brent R. Patterson, and Joseph M. Northrup. Harvest and density-dependent predation drive long-term population decline in a northern ungulate. Ecological Applications.

Appendix S3

The full specification for the population model took the following form:

$$y_{tj} \sim \text{Poisson}(\lambda_{tj})$$

$$\log(\lambda_{tj}) = \alpha_j + \log(\exp(\mu_{t+1j}) + E_{tj}) + \boldsymbol{\vartheta_j z_{tj}} + \eta_{tj} \quad \text{(S1)}$$

$$\mu_{tj} \sim \begin{cases} \text{normal}\left(\log(N_{tj}), \sigma^2_{pro_j}\right) & w = 0 \\ \text{normal}\left(\log(\hat{N}_{tj}), \left(\frac{\hat{\sigma}_{tj}}{\hat{N}_{tj}}\right)^2\right) & w = 1 \end{cases} \quad \text{(S2)}$$

$$N_{tj} = exp\left(\mu_{t-1j} + r_{max} + b_j \times \mu_{t-1j} + \beta_{predators_j} p_{tj} + \boldsymbol{\beta_j x_{tj}}\right) - E_{t-1j} \quad \text{(S3)}$$

$$E_{tj} = \sum_{k=1}^{K} E_{tjk}$$

$$E_{tjk} = E_{o_{tjk}} + E_{est_{tjk}}$$

$$E_{est_{tjk}} \sim \text{binomial}(n_{tjk}, s_{tjk})$$

$$\alpha_j \sim \text{normal}(\mu_\alpha, \sigma^2_\alpha)$$

$$\boldsymbol{\vartheta_j} \sim \text{normal}(\boldsymbol{\mu_\vartheta}, \boldsymbol{\sigma^2_\vartheta I})$$

$$\eta_{tj} \sim \text{normal}(0, \sigma^2_{obs})$$

$$\sigma^2_{pro_j} \sim \text{inverse gamma}(0.001, 0.001)$$

$$r_{max} \sim \text{normal}(0.304, 0.0064)$$

$$b_j \sim \text{normal}(\mu_b, \sigma^2_b)$$

$$\beta_{predators_j} = \beta_{canids_j} + \beta_{bears} Bear\ Density_j$$

$$\boldsymbol{\beta_j} \sim \text{normal}(\boldsymbol{\mu_\beta}, \boldsymbol{\sigma^2_\beta} I)$$

$$\beta_{canids_j} \sim \text{normal}(0,1)$$

$$\beta_{bears} \sim \text{normal}(0,1)$$

$$\sigma^2_{obs} \sim \text{inverse gamma}(0.001, 0.001)$$

$$\mu_\alpha \sim \text{normal}(0,1)$$

$$\sigma^2_\alpha \sim \text{inverse gamma}(0.001, 0.001)$$

$$\boldsymbol{\mu_\vartheta} \sim \text{normal}(\mathbf{0}, \mathbf{1}\boldsymbol{I})$$

$$\boldsymbol{\sigma^2_\vartheta} \sim \text{inverse gamma}(0.001, 0.001)$$

$$\mu_b \sim \text{normal}(0,1)\text{T}(-2,2)$$

$$\sigma^2_b \sim \text{inverse gamma}(0.001, 0.001)$$

$$\boldsymbol{\mu_\beta} \sim \text{normal}(\mathbf{0}, \mathbf{1}\boldsymbol{I})$$

$$\sigma^2_{\beta_i} \sim \text{inverse gamma}(0.001, 0.001)$$

Moose were observed by hunters (Eq. S1), y_{tj} represented the number of moose seen in year t in WMU j. α_j represented the mean log proportion of the true preharvest population of moose ($\mu_{t+1j} + E_{tj}$) seen by hunters in year t in WMU j. Where μ_{t+1j} was the log true postharvest number of moose in the upcoming winter months and E_{tj} represented the estimated number of moose harvested when hunters observed moose during the preceding fall months. $\boldsymbol{\vartheta_j}$ were WMU-specific observation coefficients for a vector of covariates $\boldsymbol{z_{tj}}$, which included the total number of days hunters reported they hunted and a fall snow depth index during year t in WMU j η_{tj} was observation level error that allowed for overdispersion.

In Eq. S2, in years where there were not aerial inventories (w = 0), the mean number of moose was estimated from the Gomppertz population model (Eq. S3). We assumed that the natural log of N_{tj} arose from a normal distribution with Gaussian process error $\sigma^2_{pro_j}$. In years where there were aerial surveys (w = 1), the mean number of moose was estimated from the aerial surveys. We assumed that the natural log of $\widehat{N}_{tj}$ arose from a normal distribution with measurement error $\hat{\sigma}_{tj}$ derived from the moose aerial survey counts. In the Gompertz model, (Eq. S3), r_{max} was the intrinsic rate of increase and b_j was the density-dependent effect for WMU j. $\beta_{predators_j}$ was the combined effect of canid and bear predators and p_{tj} was the standardized number of canids seen in WMU j during year t. $\boldsymbol{\beta_j}$ were coefficients for covariates $\boldsymbol{x_{tj}}$ that included the number of deer seen and interaction between moose abundance and canids seen in year t and WMU j. β_{canids_j} was the mean effect of canids and β_{bears} was the additive effect of $Bear\ Density_j$ on the effect of predators.

Finally, E_{tj} was estimated as the sum of $E_{o_{tjk}}$, which was the number of moose harvested by hunters that submitted surveys and $E_{est_{tjk}}$, the additional number estimated to have been harvested by hunters that did not submit surveys for WMU j during year t and for sex-age class

k. The number of animals harvested from non-reporting hunters was estimated from the total number of non-reporting hunters, N_{tjk} and the empirical success rate of reporting hunters, S_{tjk}.
